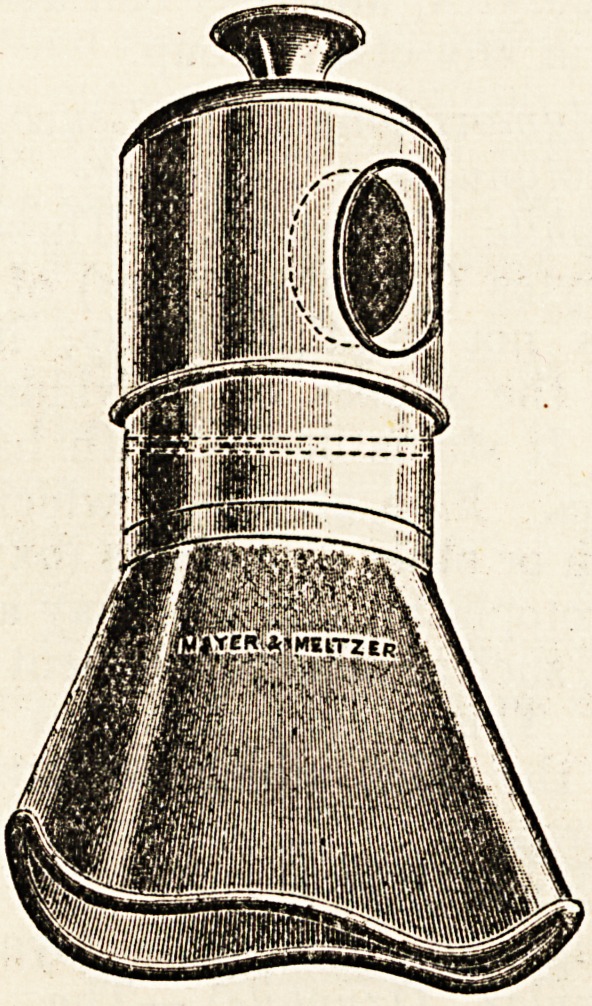# An All-Metal Inhaler

**Published:** 1910-07-09

**Authors:** E. Wilson Hird

**Affiliations:** House Surgeon to the Ear and Throat, Opthalmic and Gynæcological Departments, and late House Surgeon, General Hospital, Birmingham.


### Resident Medical Officers' Department.

### AN ALL-METAL INHALER.

By E. Wilson Hird, M.R.C.S., L.R.C.P.Lond., House Surgeon to the Ear and Throat, Opthalmic and Gynæcological Departments, and late House Surgeon, General Hospital, Birmingham.

The apparatus consists of three parts: The face-piece, anæsthetic chamber, and a metal cap.

The metal cap fits on to the anæsthetic chamber and has a large aperture on the side. This aperture, when the cap is in position, fits exactly over a similar opening in the anæsthetic chamber. By rotating this cap the strength of the vapour administered can be increased or diminished as desired. In the dome of the cap there is a small permanent airway. The anæsthetic chamber is a hollow metal cylinder open at both ends; one end receives the face-piece, the other is in contact with the dome of the metal cap. It is loosely packed with coarse sponge or gauze, maintained in position by a single metal bar running across the middle of the lower orifice. The face-piece fits into the anæsthetic chamber by bayonet joints; it is so moulded as to fit closely the curves of the average face. Very little anæsthetic is needed to induce or maintain anæsthesia.

The following advantages are claimed for this inhaler. There is an adjustable airway by means of which the strength of the vapour administered can be regulated. It is economical, as there is no overflow of the anæsthetic from the sponge or gauze. It is very easy to handle and is perfectly balanced; with the largest face-piece it only measures 6 inches in height.

By having three different-sized face-pieces a perfect fit is obtained. The bore of the inhaler is large and uniform throughout. I have found it particularly useful in the administration of the $E_2C_1$ ether sequence to children. It is easily taken to pieces for cleaning and sterilisation. A bag can be attached for the administration of ethyl chloride as a preliminary to ether. The inhaler was made for me by Messrs. Mayer and Meltzer, 171 Great Portland Street.

## Figures and Tables

**Figure f1:**